# The Role of Gulls as Reservoirs of Antibiotic Resistance in Aquatic Environments: A Scoping Review

**DOI:** 10.3389/fmicb.2021.703886

**Published:** 2021-07-23

**Authors:** Danae Zeballos-Gross, Zulma Rojas-Sereno, Marília Salgado-Caxito, Patricia Poeta, Carmen Torres, Julio A. Benavides

**Affiliations:** ^1^Departamento de Ecología y Biodiversidad, Facultad de Ciencias de la Vida, Universidad Andrés Bello, Santiago, Chile; ^2^Facultad de Ciencias de la Vida, Centro de Investigación para la Sustentabilidad, Universidad Andrés Bello, Santiago, Chile; ^3^Millennium Initiative for Collaborative Research on Bacterial Resistance (MICROB-R), Santiago, Chile; ^4^Escuela de Medicina Veterinaria, Facultad de Agronomía e Ingeniería Forestal, Facultad de Ciencias Biológicas y Facultad de Medicina, Pontificia Universidad Católica de Chile, Santiago, Chile; ^5^Department of Animal Production and Preventive Veterinary Medicine, School of Veterinary Medicine and Animal Science, São Paulo State University (UNESP), Botucatu, Brazil; ^6^Microbiology and Antibiotic Resistance Team (MicroART), Department of Veterinary Sciences, University of Trás-os-Montes and Alto Douro (UTAD), Vila Real, Portugal; ^7^Associated Laboratory for Green Chemistry (LAQV-REQUIMTE), University NOVA of Lisbon, Lisbon, Portugal; ^8^Veterinary and Animal Research Centre, Associate Laboratory for Animal and Veterinary Science (AL4AnimalS), University of Trás-os-Montes and Alto Douro (UTAD), Vila Real, Portugal; ^9^Área Bioquímica y Biología Molecular, Universidad de La Rioja, Logroño, Spain

**Keywords:** marine birds, One Health, seagulls, wildlife, bacteria, antimicrobial resistance, AMR, ESBL

## Abstract

The role of wildlife with long-range dispersal such as gulls in the global dissemination of antimicrobial resistance (AMR) across natural and anthropogenic aquatic environments remains poorly understood. Antibiotic-resistant bacteria have been detected in resident and migratory gulls worldwide for more than a decade, suggesting gulls as either sentinels of AMR pollution from anthropogenic sources or independent reservoirs that could maintain and disperse AMR across aquatic environments. However, confirming either of these roles remains challenging and incomplete. In this review, we present current knowledge on the geographic regions where AMR has been detected in gulls, the molecular characterization of resistance genes, and the evidence supporting the capacity of gulls to disperse AMR across regions or countries. We identify several limitations of current research to assess the role of gulls in the spread of AMR including most studies not identifying the source of AMR, few studies comparing bacteria isolated in gulls with other wild or domestic species, and almost no study performing longitudinal sampling over a large period of time to assess the maintenance and dispersion of AMR by gulls within and across regions. We suggest future research required to confirm the role of gulls in the global dispersion of AMR including the standardization of sampling protocols, longitudinal sampling using advanced satellite tracking, and whole-genome sequencing typing. Finally, we discuss the public health implications of the spread of AMR by gulls and potential solutions to limit its spread in aquatic environments.

## Introduction

Antimicrobial resistance (AMR) is a major global health challenge affecting human, animal, and environmental health (FAO and WHO, 2019; WHO, 2019). Thus, a One Health approach is required to understand the dynamics of AMR between humans and animals (Salgado-Caxito et al., 2021). Many studies have reported the presence of antibiotic-resistant bacteria (ARB) in wild animals, highlighting their potential role in the spread of clinically important bacteria to humans and domestic animals (Wang et al., 2017; Benavides et al., 2018; Dolejska and Literak, 2019). Wildlife such as wild birds, particularly the ones living in proximity to human settings or agriculture fields, can acquire AMR from anthropogenic sources when feeding on landfills and wastewater (Nelson et al., 2008; Wang et al., 2017). Despite several reports of wild birds carrying ARB (Wang et al., 2017), their impact on the dissemination of ARB in aquatic environments remains still poorly understood.

Gulls can impact the spread of ARB of public health concern by acting either as (i) receivers of ARB or antibiotic-resistant genes (ARGs) and acting as sentinels of human environmental pollution to natural ecosystems (Guenther et al., 2011) or as (ii) reservoirs of ARBs and ARGs, capable of dispersing ARB or ARGs to different geographic locations and to other species including humans and domestic animals. In particular, the migratory capacity of several gull species such as the Franklin’s gull (*Leucophaeus pipixcan*), migrating across America from Canada to Chile, could result in the dissemination of ARB and ARGs over extensive geographic areas, dispersing AMR from regions with high levels of AMR to less affected areas (Báez et al., 2015; Dolejska and Literak, 2019). Gulls are also present in most urban and rural environments, and their feces are extensively dispersed in the environment (Bonnedahl and Järhult, 2014). Several studies have detected ARGs in gulls (Oravcova et al., 2017; Ahlstrom et al., 2019b; Haenni et al., 2020). In particular, AMR has been detected in several species of seagulls, which have large breeding distributions in urban areas and feed on human waste (Bonnedahl et al., 2015; Stedt et al., 2015; Ahlstrom et al., 2019a). Thus, gulls have been suggested as potential reservoirs of ARB and ARGs, although evidence proving their role as reservoirs has not been provided (Radhouani et al., 2010; Aberkane et al., 2015; Merkeviciene et al., 2018).

In this scoping review, we summarized the current knowledge regarding the global dissemination of ARB and ARGs among gulls and assess whether there is evidence supporting the assumption that gulls can act as reservoirs of AMR. In particular, we aim to provide a comprehensive overview of the geographic location where ARB and ARGs have been found in gulls, the gull and bacteria species involved, as well as the antibiotic families and genes detected. To discuss the public health implications of gulls, we summarized whether bacteria of critical importance according to WHO have been detected in gulls. We also assessed the number of publications that had either identified the origin of AMR found in gulls or tested and concluded that gulls can disperse AMR across the landscape or to other species. Based on this current evidence, we discussed several recommendations aiming to improve our understanding of the role of gulls in the dissemination of AMR.

## Materials and Methods

We performed a scoping review following the Preferred Reporting Items for Systematic Reviews and Meta-Analyses extension for Scoping Reviews (PRISMA-ScR) checklist (Tricco et al., 2018; Supplementary Table 1). All authors defined research questions, objectives, search strategy, and inclusion/exclusion criteria through previous discussions.

### Search Strategy

The search was performed in PubMed, Scopus, and Web of Science databases using three general queries: (antibiotic resist^∗^ OR antimicrobial resist^∗^), (*Escherichia* OR *Klebsiella* OR *Staphylococcus* OR *Enterococcus* OR *Enterobacter*^∗^ OR *Salmonella* OR *Pseudomonas*), and (bacteria). Each of them was merged with (marine bird^∗^ OR aquatic bird^∗^ OR gull^∗^ OR *Larus*). Details of the search strategy are available as an additional file (Supplementary Table 2). Visualization, duplicate removal, and storing collected data were performed in Microsoft Excel.

### Eligibility Criteria

We aimed to identify peer-reviewed studies on AMR in different wild species of gulls (i.e., seagulls) showing the presence and/or potential transmission of ARB and ARGs. Thus, we included only studies providing at least one of the following information: (i) wild gull species where ARB was recovered, (ii) phenotypic resistance to specific antibiotics in bacteria isolated from gulls, and/or (iii) ARGs identified in bacteria isolated from gulls. There were no restrictions related to the year of publication or geographical location. Any type of reviews or studies including *in vivo* experiments, samples of gulls from rehabilitation centers, or containing previously published data were excluded. Details of all inclusion and exclusion criteria are provided in Supplementary Table 3.

### Identification and Screening of Articles

**FIGURE 1:**
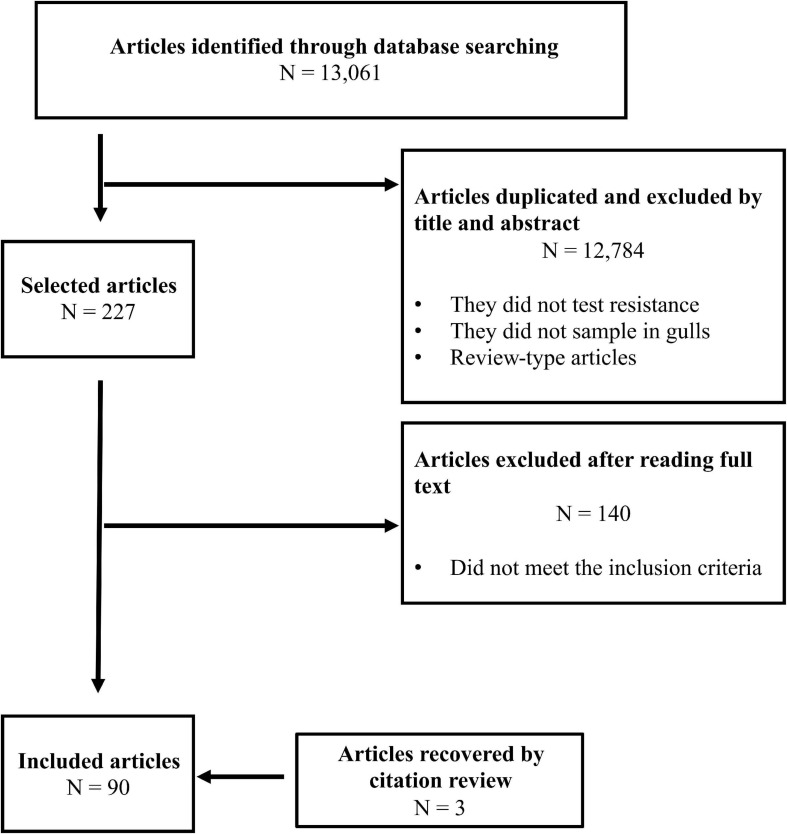
Article search flow diagram.

After the removal of duplicates, we identified a total of 3,475 articles published from 1964 to January 2021, including 3 additional references that were identified from reading these papers. Pre-selection by title and abstract reduced to 227 articles for full-text analysis, and 90 fulfilled the preestablished criteria and were included in the final analysis (Figure 1). The remaining 140 articles did not fit our inclusion criteria as they did not include gull samples, did not present AMR information/data, data from gulls were previously published, the study included experimental infection, the study was performed on captive gulls or in rehabilitation centers, the study sampling was conducted postmortem, or the full text of the article was not available.

## Data Extraction

Extracted data were independently performed by three authors (DZG, MSC, and ZRS) and verified by other authors. Disagreements were resolved through discussion. The obtained data were entered into a Microsoft Excel template adapted from a previous study (Supplementary Table 4; Salgado-Caxito et al., 2021). This file included the title of the article, authorship, year of publication, gull species included in the study and whether the species was migratory or not, the number of sampled individuals, the bacteria species studied, the number of recovered isolates, the antimicrobial susceptibility tests performed, the name and family of the antibiotics tested, and the molecular typing used (i.e., PCR, sequencing, and whole-genome sequencing) when available. To assess the current knowledge on the role of gulls as reservoirs of ARB or ARGs, we also specifically extracted from studies (i) whether the study compared gulls to other animals in the area; (ii) whether the study identified the origin (e.g., anthropogenic source) of the ARB or ARGs found; (iii) if gulls were sampled more than once, particularly in both areas of migration (origin and destination); (iv) if molecular typing of ARB was performed; and (iv) if an individual follow-up and sampling of gulls were performed, along with the method used.

### Statistical Analysis

We estimated the proportion of studies filling a given criteria (e.g., studies identifying the presence of ARGs or the origin of ARB) using R. 3.1.6 (R Development Core Team).

## Results

### Geographic Locations and Gull and Bacteria Species Studied

**FIGURE 2:**
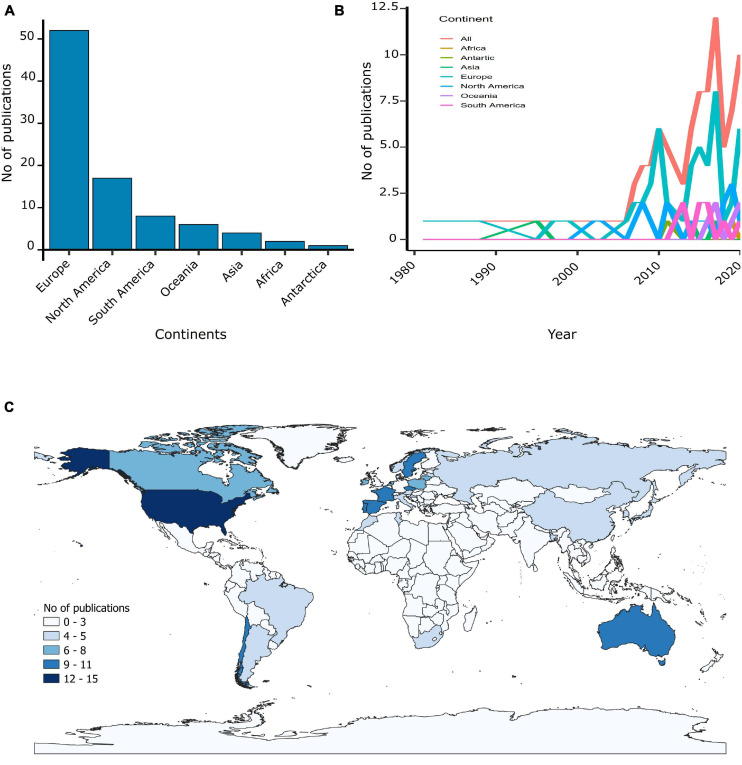
Geographical locations of the AMR studies found in gulls. **(A)** Number of publications per continents. **(B)** Number of publications per continent over the 1980–2020 period. **(C)** Number of publications of AMR in gulls per country in gradient.

Our scoping review identified 90 articles published between 1981 and 2020, although only 22% of these studies were published before 2010. The number of studies published on gulls increased from 1 in 1981 to 10 per year in 2020 and peaked in 2017 with 12 articles (Figure 2B). Studies were conducted in gulls from all five continents, but the majority of publications were made in Europe (58%) followed by North America (19%) (Figure 2A). Studies were conducted in a total of 31 countries, with high-income countries such as the United States (17%), Portugal (12%) and Spain (10%) conducting the highest number of studies (Figure 2C). In contrast, in middle- and low-income countries, few publications were conducted (Morocco, 1%; South Africa, 1%; Bangladesh, 1%).

**FIGURE 3:**
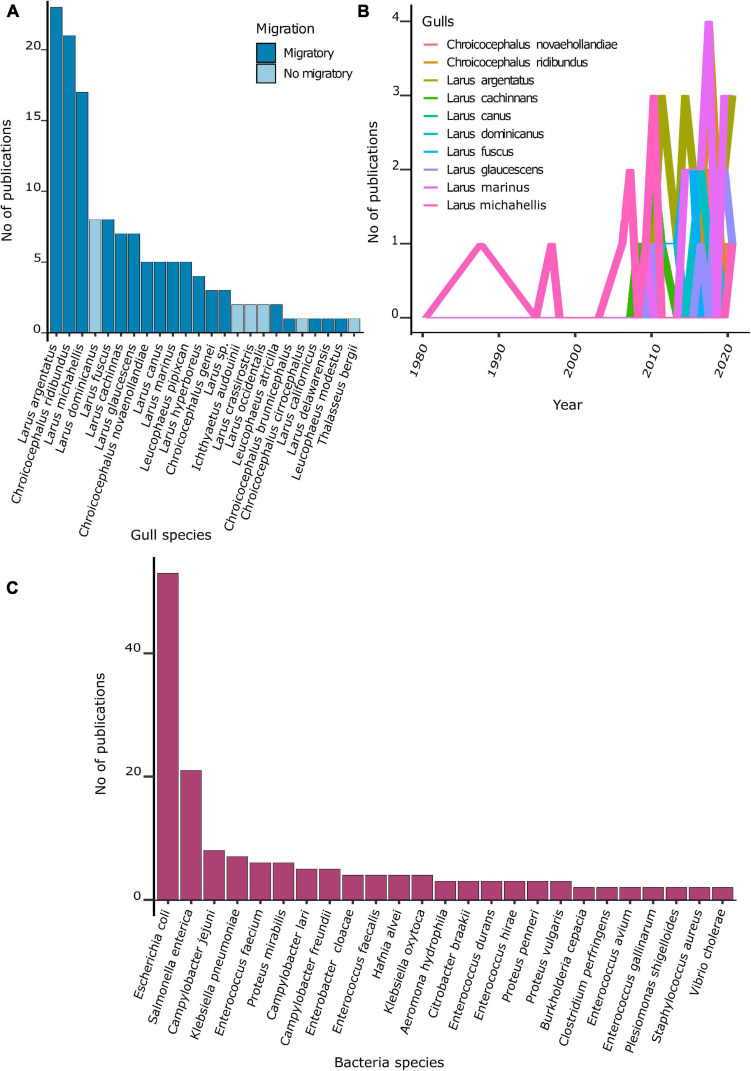
Number of publications per gull and bacteria species. **(A)** Number of publications per gull species. **(B)** Number of publications per gull species over the 1980–2020 period. **(C)** Number of publications per bacteria species.

From 100 species of gulls known (IUCN, 2021), ARB or ARGs were recovered from 23 species. Most gulls studied (74%) were migratory species. The number of studies per gull species was highly heterogeneous (Figure 3B). The majority of studies focused on the herring gull (*Larus argentatus*, 26%), followed by the laughing gull (*Chroicocephalus ridibundus*, 23%) and the yellow-legged gull (*Larus michahellis*, 19%) (Figure 3A). These three species are widely distributed in the northern hemisphere.

Among the 90 studies, 49 ARB species were recovered from gulls. Most studies focused on *Escherichia coli* (59%), *Salmonella enterica* (23%), *Campylobacter jejuni* (8%), and *Klebsiella pneumoniae* (8%) (Figure 3C). The temporal trend of publications showed that after 2008, most studies have focused on *E. coli*.

### Antibiotic Susceptibility in Bacteria From Gulls

**FIGURE 4:**
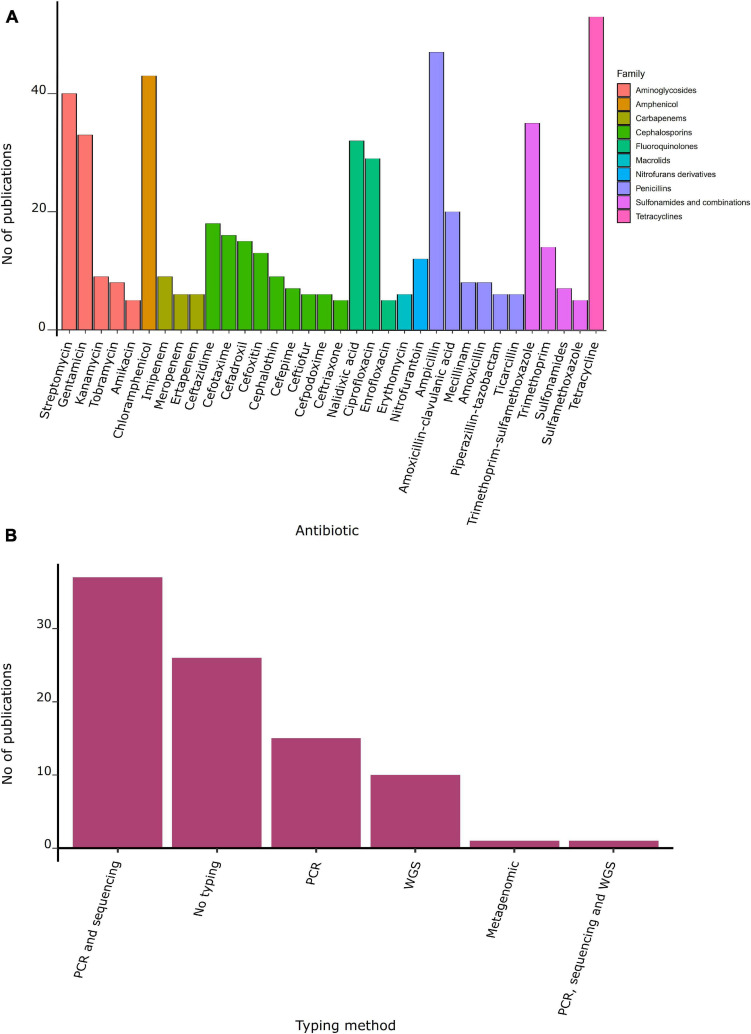
Antibiotic resistance and molecular method used. **(A)** Number of publications by family of antibiotic. **(B)** Number of publications by typing method.

Screening of ARB using selective media supplemented with antibiotics before antimicrobial susceptibility tests was performed in 43% of studies. Seventeen percent of studies performed antibiotic susceptibility tests after isolation in non-supplemented media. The remaining 40% of the studies did not present the methodology for recovering isolates. Forty-one publications had information about the number of positive individuals, and 68% of those studies were conducted in Europe. The highest proportion of animals harboring bacteria resistant to at least one antibiotic (referred as positive animals) was estimated in one study in Africa (70%) that included less than 50 individuals. The highest proportion of positive gulls was observed among *Larus dominicanus* (100%), while *Proteus mirabilis* showed the highest proportion of positive individuals (27.9%) (Table 1). Given the high heterogeneity in susceptibility methods and antibiotics tested, a comparison of ARB prevalence across studies, defined as the number of positive individuals over the total of sampled animals, could not be performed. Regarding the methodology used to test susceptibility, 68% of studies confirmed phenotypic resistance using the disk diffusion method (CLSI, 2018). Overall, resistance to 79 antibiotic agents from 21 families was tested (Supplementary Table 5), including antibiotics used in human medicine such as beta-lactams (i.e., penicillin, cephalosporins, and carbapenems), tetracyclines, fluoroquinolones, sulfonamides, aminoglycosides, nitrofurans, macrolides, monobactam, polypeptides, glycopeptides, and lincosamides. More than 50% of studies reported at least one bacterial isolate resistant to tetracycline (58%) and ampicillin (52%), followed by chloramphenicol (47%), streptomycin (44%), trimethoprim–sulfamethoxazole (38%), gentamicin (36%), nalidixic acid (35%), and ciprofloxacin (32%) (Figure 4A). In particular, broad-spectrum beta-lactams used in human medicine such as amoxicillin with clavulanic acid and ceftazidime were reported in 20% of publications.

**TABLE 1:** AMR bacteria detected in gulls by continent and gull species between 1981 and 2020.

Category	Description	Publications		No. of individuals		% of positive animals
		No.	%	Positives*	Total	
Continent	Africa	1	2.4	28	40	70.0
	Europe	28	68.3	919	6,375	14.4
	North America	5	12.2	50	1,310	3.8
	Oceania	3	7.3	57	1,108	5.1
	South America	4	9.8	164	832	19.7
	*Total*	*41*		*1,218*	*9,665*	*12.6*
Gull species	*Chroicocephalus novaehollandiae*	2	4.9	4	1,008	0.4
	*Chroicocephalus ridibundus*	2	4.9	16	1,025	1.6
	*Larus argentatus*	4	9.8	77	343	22.5
	*Larus audouinii*	1	2.4	27	111	24.3
	*Larus dominicanus*	1	2.4	10	10	100.0
	*Larus hyperboreus*	1	2.4	2	15	13.3
	*Larus michahellis*	6	14.6	260	814	31.9
	*Larus ridibundus*	6	14.6	161	2,718	5.9
	*Leucophaeus pipixcan*	1	2.4	91	124	73.4
	*Larus delawarensis*	1	2.4	2	32	6.3
	More than one species	16	39.0	568	3,465	16.4
	*Total*	*41*				
Bacteria species	*Acinetobacter baumannii*	1	2.4	2	741	0.3
	*Campylobacter* spp.	2	4.9	26	151	17.2
	*Enterobacter cloacae*	1	2.4	2	15	13.3
	*Escherichia coli*	21	51.2	805	3,395	23.7
	*Proteus mirabilis*	2	4.9	98	351	27.9
	*Salmonella enterica*	9	22.0	130	3,327	3.9
	More than one species	5	12.2	155	1,685	9.2
	*Total*	*41*				

*Positive individuals represent an individual where at least one resistant bacteria to any antibiotic researched in the study was obtained.

Among the four antimicrobial-resistant pathogens considered as a “critical priority” by the WHO (WHO, 2017), all were tested at least once in the reviewed studies. Third-generation cephalosporin-resistant Enterobacterales from gulls were the most reported (41%), followed by carbapenem-resistant Enterobacterales (10%) (Table 2). Among “high-priority” pathogens, *Campylobacter* spp. (6%) and *Salmonella* spp. (8%) both resistant to fluoroquinolones were the most identified. No “medium-priority” pathogen has been recovered from gulls.

**TABLE 2:** ARB of the “Global Priority Pathogens” list of the World Health Organization isolated from gulls reported between 1981 and 2020.

Priority category	Pathogens	Antibiotic resistance	No. of publications	Gull species	References
Critical	*Acinetobacter baumannii*	Carbapenem-resistant	1% (1/90)	*C. ridibundus*	Łopińska et al., 2020
	*Pseudomonas aeruginosa*	Carbapenem-resistant	0% (0/90)	−	−
	Enterobacterales*	Carbapenem-resistant	10% (9/90)	*L. glaucescens, L. argentatus, L. hyperboreus, C. novaehollandiae, L. michahellis, C. genei*	Papagiannitsis et al., 2017; Vergara et al., 2017; Vittecoq et al., 2017; Ahlstrom et al., 2018, 2019a,c; Barguigua et al., 2019; Mukerji et al., 2019; Aires-De-sousa et al., 2020
	Enterobacterales*	Third generation cephalosporin-resistant	41% (37/90)	*L. glaucescens, L. argentatus, L. hyperboreus, L. fuscus, L. michahellis, C. novaehollandiae, L. glaucescens, C. ridibundus, L. marinus, L. canus, L. cachinnans, L. dominicanus, Leucophaeus pipixcan, C. brunnicephalus, L. atricilla*	Poeta et al., 2008; Bonnedahl et al., 2009, 2014; Rose et al., 2009; Hernandez et al., 2010; Simões et al., 2010; Veldman et al., 2013; Hasan et al., 2014; Stedt et al., 2015; Alcalá et al., 2016; Aberkane et al., 2016, 2017; Atterby et al., 2016, 2017; Dolejska et al., 2016; Liakopoulos et al., 2016; Merkeviciene et al., 2017, 2018; Papagiannitsis et al., 2017; Troxler et al., 2017; Vergara et al., 2017; Ahlstrom et al., 2018, 2019a,b, 2021; Mukerji et al., 2019, 2020; Ngaiganam et al., 2019; Aires-De-sousa et al., 2020; Haenni et al., 2020; Zendri et al., 2020
High	*Enterococcus faecium*	Vancomycin-resistant	3% (3/90)	*Chroicocephalus novaehollandiae, L. cachinnans*	Radhouani et al., 2010; Bonnedahl et al., 2014; Oravcova et al., 2017
	*Staphylococcus aureus*	Methicillin-resistant	2% (2/90)	*L. argentatus*	Merkeviciene et al., 2017; Aires-De-sousa et al., 2020
	*Staphylococcus aureus*	Vancomycin-intermediate	0% (0/90)	−	−
	*Staphylococcus aureus*	Vancomycin-resistant	0% (0/90)	−	−
	*Helicobacter pylori*	Clarithromycin-resistant	0% (0/90)	−	−
	*Campylobacter* spp.	Fluoroquinolone-resistant	6% (5/90)	*L. michahellis*, *L. audouinii*, *C. ridibundus*, *L. dominicanus*, *Thalasseus bergii*	Merkeviciene et al., 2017; Migura-Garcia et al., 2017; Moré et al., 2017; Troxler et al., 2017; Antilles et al., 2021
	*Salmonella* spp.	Fluoroquinolone-resistant	8% (7/90)	*L. michahellis*, *L. audouinii, L. dominicanus*, C. *novaehollandiae*, *C. ridibundus*, *Leucophaeus pipixcan, Leucophaeus modestus*	Fresno et al., 2013; Antilles et al., 2015, 2021; Retamal et al., 2015; Masarikova et al., 2016; Cummins et al., 2020; Tardone et al., 2020
	*Neisseria gonorrhoeae*	Third generation cephalosporin-resistant	0% (0/90)	−	−
	*Neisseria gonorrhoeae*	Fluoroquinolone-resistant	0% (0/90)	−	−
Medium	*Streptococcus pneumoniae*	Penicillin-non-susceptible	0% (0/90)	−	−
	*Haemophilus influenzae*	Ampicillin-resistant	0% (0/90)	−	−
	*Shigella* spp.	Fluoroquinolone-resistant	0% (0/90)	−	−

*Klebsiella pneumoniae, Escherichia coli, Enterobacter spp., Serratia spp., Proteus spp., Providencia spp., and Morganella spp.

### Molecular Characterization of ARGs in Gulls

ARGs were reported in 70% of studies conducted in gulls (Tables 3, 4). Mobile genetic elements (MGE) were identified in 43% of studies, with 35 studies confirming that ARGs were inserted on an MGE. Sixteen percent of studies detected ARGs using PCR alone, or in combination with sequencing (43%). Only 8% of studies characterized bacteria by whole-genome sequencing, and one study used a metagenomic approach (Figure 4B).

**TABLE 3:** Beta-lactamases genes identified in isolates from gulls reported between 1981 and 2020.

Continent	AmpC	CP	ESBL	Others^a^	References
Africa	NR	bla_OXA–48_	NR	NR	Barguigua et al., 2019
Antarctica	NR	NR	NR	NR	−
Asia	NR	NR	*bla*_CTX__–__M__–__14_, *bla*_CTX__–__M__–__15_, *bla*_CTX__–__M__–__55_, *bla*_CTX__–__M__–__79_	NR	Hasan et al., 2014
Europe	*bla*_CMY__–__2_, *bla*_CMY_, *bla*_DHA__–__1_, *bla*_ACT__–__14_, *bla*_ACT__–__15_, *bla*_ACT__–__23_	*bla*_OXA__–__48_, *bla*_OXA__–__181_, *bla*_KPC__–__2_, *bla*_KPC__–__3_, *bla*_OXA__–__71_, *bla*_OXA__–__208_, *bla*_VIM__–__1_, *bla*_VIM__–__4_	*bla*_CTX__–__M__–__15_, *bla*_CTX__–__M__–__55_, *bla*_SHV__–__2_, *bla*_CTX__–__M__–__1_, *bla*_CTX__–__M__–__14_, *bla*_CTX__–__M__–__27_, *bla*_CTX__–__M__–__9_, *bla*_SHV__–__12_, *bla*_PER_, *bla*_CTX__–__M__–__32_, *bla*_CTX__–__M_, *bla*_TEM__–__84_, *bla*_CTX__–__M__–__2_, *bla*_CTX__–__M__–__8_, *bla*_CTX__–__M__–__3_, *bla*_TEM__–__5__2C_, *bla*_TEM__–__52_, *bla*_CTX__–__M__–__1__4a_, *bla*_PSE__–__1_	*bla*_TEM_, *bla*_OXA__–__1__–__like_, *bla*_TEM__–__1_, *bla*_SHV_, *bla*_OXA__–__1_, *bla*_OXA__–__3_, *bla*_OXA__–__5_, *bla*_TEM__–1b_	Čížek et al., 2007; Dolejska et al., 2007; Poeta et al., 2008; Bonnedahl et al., 2009, 2010; Dolejská et al., 2009; Radhouani et al., 2009; Hernandez et al., 2010; Literak et al., 2010, 2014; Simões et al., 2010; Wallensten et al., 2011; Veldman et al., 2013; Vredenburg et al., 2014; Aberkane et al., 2015, 2016, 2017; Antilles et al., 2015; Stedt et al., 2015; Varela et al., 2015; Carroll et al., 2015; Masarikova et al., 2016; Alcalá et al., 2016; Atterby et al., 2017; Merkeviciene et al., 2017, 2018; Vergara et al., 2017; Vittecoq et al., 2017
North America	*bla*_ampC_, *bla*_CMY__–__2_, *bla*_CMY__–__61_, *bla*_DHA__–__1_, *bla*_CMY_	*bla*_KPC__–__2_, *bla*_OXA__–__48_, *bla*_OXA__–__9_, *bla*_CARB__–__1_, *bla*_CARB__–__2_, *bla*_CARB_	*bla*_CTX__–__M_, *bla*_CTX__–__M__–__1_, *bla*_CTX__–__M__–__14_, *bla*_CTX__–__M__–__15_, *bla*_CTX__–__M__–__27_, *bla*_CTX__–__M__–__32_, *bla*_CTX__–__M__–__3_, *bla*_CTX__–__M__–__55_, *bla*_CTX__–__M__–__65_, *bla*_CTX__–__M__–__8_, *bla*_TEM__–__141_, *bla*_TEM__–__52_, *bla*_TEM__–__19_, *bla*_TEM__–__206_, *bla*_TEM__–__214_, *bla*_SHV__–__12_, *bla*_SHV__–__2_, *bla*_SHV__–2A_, *bla*_SHV__–__11_, *bla*_SHV__–__14_	*bla*_TEM__–1A_, *bla*_TEM__–1B_, *bla*_TEM__–1C_, *bla*_TEM__–1D_, *bla*_OXA__–__1_, *bla*_OXA__–__466_, *bla*_ampH_, *bla*_ampC__2_, *bla*_mrdA_, *bla*_ampC__1_, *bla*_TEM__–__1_, *bla*_SHV__–__1_, *bla*_TEM_	Alroy and Ellis, 2011; Martiny et al., 2011; Bonnedahl et al., 2014, 2015; Atterby et al., 2016; Ahlstrom et al., 2018, 2019a,b, 2021; Gomez-Alvarez et al., 2019
Oceania	*bla*_CMY__–__2_, *bla*_CMY__–__13_, *bla*_CMY__–__42_, *bla*_CMY__–__60_	*bla*_OXA__–__48_, *bla*_IMP__–__4_, *bla*_IMP__–__38_	*bla*_CTX__–__M__–__15_, *bla*_CTX__–__M__–__27_, *bla*_CTX__–__M__–__14_, *bla*_CTX__–__M__–__3_, *bla*_CTX__–__M__–__55_, *bla*_CTX__–__M__–__11_, *bla*_CTX__–__M__–__24_	*bla*_TEM__–__1_, *bla*_LAP__–__2_, *bla*_TEM_, *bla*_OXA__–__1_, *bla*_SHV_, *bla*_OXA__–__1_, *bla*_TEM__–__1_	Dolejska et al., 2016; Papagiannitsis et al., 2017; Mukerji et al., 2019, 2020; Cummins et al., 2020
South America	NR	NR	*bla*_CTX__–__M__–__1_, *bla*_CTX__–__M__–__2_, *bla*_CTX__–__M__–__14_, *bla*_SHV__–2A_, *bla*_SHV__–__2_, *bla*_CTX__–__M__–__15_, *bla*_CTX__–__M__–__22_, *bla*_CTX__–__M__–__3_, *bla*_TEM__–__40_, *bla*_TEM__–__198_, *bla*_SHV__–__12_	*bla*_TEM__–__1_	Hernandez et al., 2013; Báez et al., 2015; Liakopoulos et al., 2016

*AmpC, cephalosporinases; CP, carbapenemases; NR, not reported.*

*^*a*^Corresponds to beta-lactamases that are not classified as ESBL, AmpC, or CP.*

**TABLE 4:** AMR genes identified in isolates from gulls reported between 1981 and 2020.

Continent	FQ	POLY	TET	AMG	CHL	SUL	TMP	MAC	STR	GLY	FOS	RIF	References
Africa	*aac(6′)-Ib-cr*, *qnrS1*, *qnrB1*	NR	NR	NR	NR	NR	NR	NR	NR	NR	NR	NR	Barguigua et al., 2019
Antarctica	NR	NR	NR	NR	NR	NR	NR	NR	NR	NR	NR	NR	−
Asia	NR	NR	NR	NR	NR	NR	NR	NR	NR	NR	NR	NR	−
Europe	*aac(6′)-Ib-cr*, *qnrB*, *gyrA*, *parC*, *qnrA1*, *qnrS*, *qnrB1*, *qnrS1*	*mcr-9*, *mcr-1*,	*tetA*, *tetB*, *tetG*, *tetL*, *tetM*, *tetD*	*aadB*, *aadA*, *aadA1*, *aadA2*, *aadA4*, *aadA5*, *rmtB*, *armA*, *aphA1*, *aacA4*, *aac(3)II*, *strA*, *strB*, *aac(6′)-Ib*, *aph(30′)-IIIa*, *ant(6)-Ia*, *sat*, *aac(3)-IV*, *aac(6′)*, *aadA1a*	*catII*, *catA*, *catA1*, *cmlA*, *cmlA1*, *floR*, *cat*, *catB3*	*sul1*, *sul2*, *sul3*	*dfr1*, *dfr5*, *dfr7*, *dfrA16*, *dfrA1*, *dfrA12*, *dfrA14*, *dfrA17*, *dfrA7*, *dfrA15*	*ermB*	*vatE*, *vatD*	*vanA*	NR	NR	Čížek et al., 2007; Dolejska et al., 2007; Gionechetti et al., 2008; Poeta et al., 2008; Dolejská et al., 2009; Radhouani et al., 2009, 2010, 2011; Bonnedahl et al., 2009, 2010; Hernandez et al., 2010; Literak et al., 2010, 2014; Simões et al., 2010; Wallensten et al., 2011; Veldman et al., 2013; Vredenburg et al., 2014; Aberkane et al., 2015, 2016; Carroll et al., 2015; Aberkane et al., 2017; Stedt et al., 2015; Varela et al., 2015; Antilles et al., 2015; Masarikova et al., 2016; Ruzauskas and Vaskeviciute, 2016; Alcalá et al., 2016; Merkeviciene et al., 2017, 2018; Vergara et al., 2017; Vittecoq et al., 2017; Atterby et al., 2017; Ngaiganam et al., 2019; Ahlstrom et al., 2019b; Haenni et al., 2020; Łopińska et al., 2020; Zendri et al., 2020; Aires-De-sousa et al., 2020
North America	*aac(6′)-Ib-cr*, *gyrA*, *parC*, *parE*, *qnrB4*, *qnrS1*, *oqxB*, *qnrA1*, *qnrB*, *qnrA*	NR	*tetA*, *tetB*, *tetC*, *tetD*, *tetR*	*aac3, aac(3)-Iia*, *aac(3)-IId*, *aac(3)-VIa*, *aadA*, *aadA1*, *aadA2*, *aadA2b*, *aadA5*, *ant(2″)-Ia*, *aph(3″)-Ib*, *aph(3′)-Ia*, *aph(6)-Id*, *aph(3′), aac(3)-IIa*, *aph(3′)-IIa*, *strA*, *strB*	*catA1*, *catB3*, *catB4*, *cmlA1*, *floR*	*sul1*, *sul2*, *sul3*	*dfrA1*, *dfrA5*, *dfrA7*, *dfrA8*, *dfrA12*, *dfrA14*, *dfrA15*, *dfrA16*, *dfrA17*, *dfrA5*, *dfrA8*	*ermB*, *mphA*, *mphE*, *ereA*	NR	NR	*fosA3, fosA4, fosA7*	NR	Alroy and Ellis, 2011; Martiny et al., 2011; Bonnedahl et al., 2014, 2015; Atterby et al., 2016; Ahlstrom et al., 2018, 2019a,c, 2021; Gomez-Alvarez et al., 2019
Oceania	*qnrS*, *qnrB*, *qnrS1*, *qnrB4*, *qnrB6*	*mrc-1*	*tetA*, *tetM*	*strA*, *strB*, *aac(6′)-Iy*, *ant(3″)-IIa*, *aph(3′)-Ia*, *aac(3)-IId*, *aph(3′)-IIIa*, *aac(6′)aph(2″)*	*floR*	*sul2*, *sul3*	*dfrA14*	*mphA*, *ermB*	NR	*vanB*	*fosA7*	*arr-2*	Dolejska et al., 2016; Oravcova et al., 2017; Papagiannitsis et al., 2017; Mukerji et al., 2019, 2020; Cummins et al., 2020
South America	NR	NR	*tetA*	*strA*, *strB*	NR	NR	NR	NR	NR	NR	NR	NR	Hernandez et al., 2013; Báez et al., 2015; Liakopoulos et al., 2016; Toro et al., 2016

*FQ, fluoroquinolones; POLY, polypeptides; TET, tetracyclines; AMG, aminoglycosides; CHL, chloramphenicol; SUL, sulfonamides; TMP, trimethoprim; MAC, macrolides; STR, streptogramins; GLY, glycopeptides; FOS, fosfomycin; RIF, rifamycin; NR, not reported.*

Most studies detecting ARGs focused on beta-lactamase genes including extended-spectrum beta-lactamases (ESBL), AmpC-type beta-lactamases, and carbapenemases, which were identified in all continents but Antarctica (Table 3). Among these beta-lactamases, ESBL were the most identified genes, evenly distributed across continents, particularly the genotype *bla*_CTX__–__M_. Studies detected *bla*_CTX__–__M__–__14_ and *bla*_CTX__–__M__–__15_ in Asia, Europe, North and South America, and Oceania. Likewise, *bla*_CTX__–__M__–__55_ was reported in all these continents with the exception of South America. Beta-lactamases *bla*_CMY__–__2_ (AmpC) were reported in Europe, North America, and Oceania, and *bla*_OXA__–__48_ (carbapenemase) were reported in Africa, Europe, North America, and Oceania (Table 3). Of the 15 studies that found AmpC-type beta-lactamases, 7 identified that they were inserted on an MGE, 2 identified them on the bacterial core genome, 2 detected both chromosomal and acquired AmpC, and 4 studies did not identify the location of the AmpC gene. Genes conferring resistance to other antibiotics such as fluoroquinolones, aminoglycosides, sulfonamides along with trimethoprim, polypeptides, tetracyclines, chloramphenicol, macrolides, streptogramins, glycopeptides, fosfomycin, and rifamycin were reported in 59 studies (Table 4). Asia only reported beta-lactam resistance genes.

### Origin of AMR in Gulls

Only 19% of studies suggested a potential origin for the ARB or ARGs detected among gulls. Landfill (41%), places close to gulls nesting, and/or resting areas with high human density (29%), sewage effluents (29%), and contaminated water (6%) were suspected. Suspicions were based on potential contamination sources around the sampling area. However, only one study (Masarikova et al., 2016) carried out sampling to verify whether the gulls acquired the bacteria from a specific contamination source, comparing bacteria from gulls to bacteria isolated from sewage water near their nesting sites. The same AMR phenotypic profiles were obtained in both sample types, and pulsed-field gel electrophoresis (PFGE) detected the same AMR profiles in bacterial clones from wastewater and gulls.

### Evidence of Gulls Acting as Reservoirs of ARB or ARGs

Two studies (2%) tagged gulls in both the origin and final movement areas to identify whether they were capable of spreading AMR across the landscape (Palmgren et al., 2006; Ahlstrom et al., 2019a). Ahlstrom et al. (2019a) sampled individual gulls at different periods of time obtaining fecal samples at a landfill and in places where humans and seagulls gathered. Satellite telemetry was used to monitor individuals for up to 3 months, and whole-genome sequencing of bacteria was used to compare *E. coli* isolates between different locations. Their results showed that the prevalence and genetic typing of AMR isolates were highly similar between gulls and a landfill. Palmgren et al. (2006) ringed gulls and sampled 1,047 individuals for up to 3 years. This study failed to detect long-term carriage of antibiotic-resistant *Salmonella* since all positive individuals were negative during the sampling 2 months later.

## Discussion

AMR has been detected in resident and migratory gulls worldwide for more than a decade (Fenlon, 1981; Tsubokura et al., 1995; Smith et al., 2002). However, the role of gulls as reservoirs (i.e., having the capacity to disperse and transmit AMR to other species) remains unknown. Our review identified 90 studies on AMR in wild gulls. AMR has been widely detected across all continents including in 23 of 100 species of gulls (IUCN, 2021), 49 bacteria species with 9 of 13 ARB classified as critical priority for human health (WHO, 2017), ARGs from 13 classes, and 47 antibiotic types. Our results show that, with the exception of China, studies in middle- and low-income countries are rare. Similarly, most studies have focused on a few species of gulls from Europe (e.g., *L. argentatus* and *C. ridibundus*) and most on *E. coli* and *Salmonella spp*. Despite ARB and ARGs being widely detected in gulls, our analyses showed that the origin of these AMR remains unknown in 81% of studies, and only two studies followed gulls across time (for up to 3 years), but none has been able to prove that gulls were reservoirs (Palmgren et al., 2006; Ahlstrom et al., 2019a). Therefore, our review highlights the need to increase surveillance of AMR in gulls and design innovative studies aiming to assess their role as reservoirs, which can have major implications for public and conservation measures to limit the global spread of AMR in aquatic systems.

The detection of AMR in gulls across all continents, including critically important antibiotic-resistant pathogens such as ESBL and carbapenemase-producing *E. coli* and *S. enterica*, illustrates the potential of gulls to participate in the alarming global spread of AMR (Dolejska et al., 2016; Ahlstrom et al., 2019c; Aires-De-sousa et al., 2020; Cummins et al., 2020). The migratory capacity of gulls makes them an ideal host to spread ARB and ARGs across landscapes and ecosystems. For example, Ahlstrom et al. (2021) reported that gulls of the *Larus* genus, including *L. argentatus*, can migrate 3,000 km over a week, *Larus fuscus* can migrate from Europe to Africa (Kilpi and Saurola, 1984), while *Leucophaeus pipixcan* migrates from North to South America (Hernandez et al., 2013; Barbieri et al., 2016). In contrast, other gull species such as *L. dominicanus* are resident but also carry ESBL-resistant *E. coli* with ARGs genes such as *bla*_CTX__–__M_ and *bla*_SHV_ and aminoglycoside-resistant *Salmonella enteritidis* with *str* genes (Liakopoulos et al., 2016; Toro et al., 2016). Although these species might not necessarily contribute to the long-range dispersal of AMR, they could participate in local transmission to other species and humans (Vigo et al., 2011; Retamal et al., 2015; Toro et al., 2016). Overall, this review highlights that gulls are at least sentinels of ARB and ARGs spreading in the environment, calling for future research in species and countries where AMR has not yet been studied. In particular, environmental and animal health national and international authorities should consider gulls in the surveillance of AMR within the environment.

Although AMR is widely spread among gulls, there are almost no data on the origin of the observed ARB and ARGs. In fact, less than 20% of studies included in this review mentioned potential sources of AMR contamination. Given that AMR has exponentially increased with antibiotic use in humans and livestock and several gull species feed on human and agricultural waste, most studies suspect a human origin including landfills, places close to gulls nesting, and/or resting areas that have a high human density, sewage effluents, and contaminated water (Bonnedahl et al., 2014; Atterby et al., 2016; Mukerji et al., 2019; Ahlstrom et al., 2021). This is consistent with the overall assumption that wildlife becomes contaminated with AMR from anthropogenic sources in studies suggesting transmission in areas where wildlife lives and feeds (Dolejska and Literak, 2019). However, no study has fully proven the origin of AMR in gulls, and other environmental factors such as co-selection with heavy metals and microplastics can also generate AMR (Gullberg et al., 2014; Dong et al., 2021). In our review, only one study sampled a potential contamination source to identify the origin of AMR find in gulls, showing that isolates from wastewater and gulls had the same macrorestriction profiles (Masarikova et al., 2016). One possible explanation for the small number of studies trying to identify the origin of AMR in gulls could be that no standard sampling protocol or specific criteria are available to fully determine the origin. Alternatively, logistical challenges such as collecting both wildlife, domestic animals, and human environments at the same time could limit the realization of these studies. Future research could follow methodologies used by studies performed on bacteria susceptible to antibiotics and other wildlife. For example, Nelson et al. (2008) characterized *E. coli* from gulls, garbage, and sewage by ribotyping, finding isolates with > 90% similarity in the band patterns between gulls and sewage. However, this study was not included in this review because it did not test for ARB or ARGs. Similarly, other studies have simultaneously sampled domestic animals and wildlife where contact between species can be frequent (e.g., small-scale farms) to assess potential cross-species transmission of ESBL-*E. coli* (Benavides et al., 2021). Although challenging, identifying the origin of AMR in gulls is essential when planning preventive strategies to limit the spread of AMR in natural ecosystems. Seagulls are characterized by being ubiquitous in most urban and rural environments, and many of them are migratory, so it is assumed that gulls may disperse ARB and ARGs between countries or even continents. Despite this assumption, only two studies included in this review performed longitudinal samplings to test the long-term carriage of ARB or ARGs in gulls (Palmgren et al., 2006; Ahlstrom et al., 2019a), requiring further research to identify their implication as reservoirs of AMR.

The detected ARB and ARGs found in gulls have major implications for both animal and human health. *E. coli* was the most common bacterial species reported followed by *Salmonella*, similarly to other wildlife species (Vittecoq et al., 2016). Both bacterial species are important for public health and are considered a critical priority for human and animal health (Vittecoq et al., 2016; WHO, 2017). *E. coli* and *Salmonella spp.* can also be found at equilibrium as commensal bacteria, so the impact of these bacteria of gull’s health is unknown. Other reported pathogens found in gulls, such as *Campylobacter spp.* and *Klebsiella pneumoniae*, are considered as zoonotic pathogens and could represent a threat to human health. Global priority antibiotic-resistant pathogens for human and animal health were increasingly reported among gulls after 2008, particularly those considered as “critical” (WHO, 2017). Broad-spectrum antimicrobial therapies are commonly used to treat bacterial infections in both humans and animals (Bush and Jacoby, 2010). The widespread detection of third-generation cephalosporin-resistant Enterobacterales in addition to resistance to other important antimicrobials, such as carbapenems and fluoroquinolones, could compromise the effective treatment rates representing an important threat to public and veterinary health.

The higher detection of antibiotic-resistant enterobacteria could be explained by the relatively easier collection of fecal samples compared to capturing and sampling gulls to detect other pathogens (e.g., blood bacteria). Thus, the absence of other global-priority ARB in current studies such as *Pseudomonas aeruginosa* carbapenem-resistant (critical priority), *Staphylococcus aureus* vancomycin-intermediate or -resistant (high priority), and *Shigella* spp. fluoroquinolone-resistant (medium priority) could reflect a lack of research and not necessarily that these bacteria are not circulating among gulls. In fact, one study using a metagenomic approach found 31 previously undescribed ARGs, while another detected more than 70 bacterial species and 24 ARGs (Martiny et al., 2011; Merkeviciene et al., 2017). This review identified a high diversity of ARGs including those implicated in bacterial infections of humans and animals such as ESBL and carbapenemases (Bevan et al., 2017; Li et al., 2019). The presence of ARGs inserted in MGE could facilitate the spread of these resistance genes within gulls and between humans and other animals (Loayza et al., 2020). Our review also shows a wide diversity of bacterial clones and ARGs found in gulls. Whole-genome sequencing for bacterial typing was used in 13% of studies since 2011. Thus, the more widespread use of this technique in the following years could increase the detection of ARB clones and ARGs in gulls.

Our review showed that North America and Europe had the most diverse molecular diversity among ARB, which could be associated with more available molecular typing techniques compared to low-income countries. For example, ESBL- *E. coli* ST131, previously associated with nosocomial infections in humans, has been identified in gulls mainly from the United States (Bonnedahl et al., 2014; Ahlstrom et al., 2018, 2019a, 2021) and Portugal (Simões et al., 2010; Vredenburg et al., 2014; Varela et al., 2015). Despite fewer information available, ST131 has also been reported in gulls from low- and middle-income countries (LMICs) such as Bangladesh (Hasan et al., 2014). Future research should also evaluate the pathogenic potential of the detected ARB using whole-genome sequencing to detect virulence factors and other pathogenic genetic material (e.g., biofilms).

## Conclusion and Future Directions

Our review identified an increasing interest in ARB and ARGs among gulls in the last decade, although there is a considerable lack of information in LMICs, particularly regarding migratory species. Despite the widespread detection and high diversity of ARB and ARGs worldwide, there is no evidence that gulls act as reservoirs of ARB and ARGs. Furthermore, most of the studies could not demonstrate whether ARB and ARGs in gulls came from anthropogenic sources. Finally, we could not compare ARB or ARGs prevalence across studies due to their heterogeneity in the results and methodologies to assess AMR.

Knowledge gaps identified in this review can be overcome by future research. First, the use of whole-genome sequencing combined with sampling across different species could help assessing cross-species transmission between gulls and humans, domestic animals, or other wild species that do not usually interact with humans but share nesting sites with gulls (e.g., penguins). Secondly, future research should identify if environmental factors such as plastic and heavy metal pollution are also selecting ARB and ARGs in gulls independently of contact with humans. Thirdly, the clinical relevance and conservation implications of the detected ARB for gull’s health require further investigation. In particular, there is no evidence that the observed bacteria cause any pathogenicity to the studied gulls nor complicate treatment of gulls with antibiotics in rehabilitation centers. Finally, innovative techniques such as satellite tracking and collaborations across research teams in different countries where gulls migrate (e.g., Canada to Chile for the Franklin’s gulls) could help elucidate whether gulls are spreading ARB and/or ARGs across countries and continents during their migration.

## Author Contributions

JB and DZ-G: conceptualization. DZ-G: data curation. JB, DZ-G, ZR-S, and MS-C: formal analysis, investigation, and methodology. JB: funding acquisition, project administration, resources, software, supervision, validation, and visualization. DZ-G, JB, ZR-S, MS-C, CT, and PP: writing—original draft and writing—review and editing. All authors have read and agreed to the published version of the manuscript.

## Conflict of Interest

The authors declare that the research was conducted in the absence of any commercial or financial relationships that could be construed as a potential conflict of interest.

## Publisher’s Note

All claims expressed in this article are solely those of the authors and do not necessarily represent those of their affiliated organizations, or those of the publisher, the editors and the reviewers. Any product that may be evaluated in this article, or claim that may be made by its manufacturer, is not guaranteed or endorsed by the publisher.
